# Histone monoaminylation is a novel epigenetic mechanism in psychiatric disorders

**DOI:** 10.3389/fnmol.2025.1534569

**Published:** 2025-02-18

**Authors:** Jacob Peedicayil, Samuel Santhosh

**Affiliations:** Department of Pharmacology and Clinical Pharmacology, Christian Medical College, Vellore, India

**Keywords:** epigenetic, histone, monoamine, novel, psychiatry

## Introduction

Monoamines, basic nitrogenous molecules containing one amine group, include serotonin, adrenaline, noradrenaline, dopamine, and histamine. They are mainly synthesized due to decarboxylation of amino acids or by transamination and amination of ketones and aldehydes (Al-Kachak and Maze, 2023). They are important neurotransmitters in the body, especially in the central nervous system, but also in the peripheral nervous system, and the enteric nervous system.

## Protein monoaminylation

Protein monoaminylation is the covalent bonding of biogenic monoamines to glutamine residues in some proteins by a transamidation reaction. It was first described by Heinrich Waelsch's group in the 1950s (Sarkar et al., 1957; Al-Kachak and Maze, 2023). This group showed that mono-and poly-amines can be included into proteins of livers obtained from guinea pigs, mice, rats, and rabbits, with the help of calcium-dependent transglutaminases (TGMs). In mammals nine isozymes of transglutaminases have been delineated (Zhuang and Khosla, 2019). Human TGM2 is multi-functional and ubiquitous and takes part in many cellular processes such as apoptosis, development, differentiation, wound healing, and angiogenesis (Kim and Park, 2020). In the cell TGM2 is mainly located in the cytosol, but also in the nucleus, the cell surface, and extracellularly (Keillor et al., 2015). It has many functions depending on its localization and is allosterically stimulated by calcium ions and deactivated by guanosine triphosphate (Keillor et al., 2015). Human TGM2 comprises 687 amino acid residues with a four-domain structure and a molecular weight of about 70 kDa (Kim and Park, 2020). TGMs cross-link the γ-carboxamide of a glutamine residue and the ϵ-amino group of a lysine residue (Kim and Park, 2020), and catalyze the monoaminylation of glutamine residues of target proteins (Jiang et al., 2021; Al-Kachak and Maze, 2023). Since the work by Waelsch's group, many proteins including fibronectin, fibrinogen, actin, and myosin have been found to be monoaminylated (Bader, 2019).

## Histone monoaminylation

Modifications of histones are a well-established epigenetic mechanism of regulation of gene expression that can transduce environmental signals to modulate gene expression (Vanzan et al., 2023; Wu et al., 2023). Histones undergo many dynamic and reversible biochemical post-translational modifications (Kennelly et al., 2023). Histone acetylation and deacetylation are the best studied such modifications that histones undergo. Others include methylation, phosphorylation, sumoylation, ADP-ribosylation, citrullination, and proline isomerisation (Vanzan et al., 2023). Histone modifications can reduce the positive charges of histone tails and hence reduce the binding affinity of histone tails to the negatively charged DNA (Kennelly et al., 2023). Additionally, covalent modifications of histones form extra binding sites for proteins like ATP-dependent chromatin-remodeling complexes which contain subunits that specifically attach to histones with specific modifications. These complexes may enhance accessibility of neighboring DNA sequences by modifying or removing histones. The histone modifications and chromatin- remodeling complexes can synergistically act to open up gene promoters and regulatory regions, thereby permitting the binding of trans-factors, RNA polymerase II, and general transcription factors (GTFs)(Kennelly et al., 2023).

**Figure 1:**
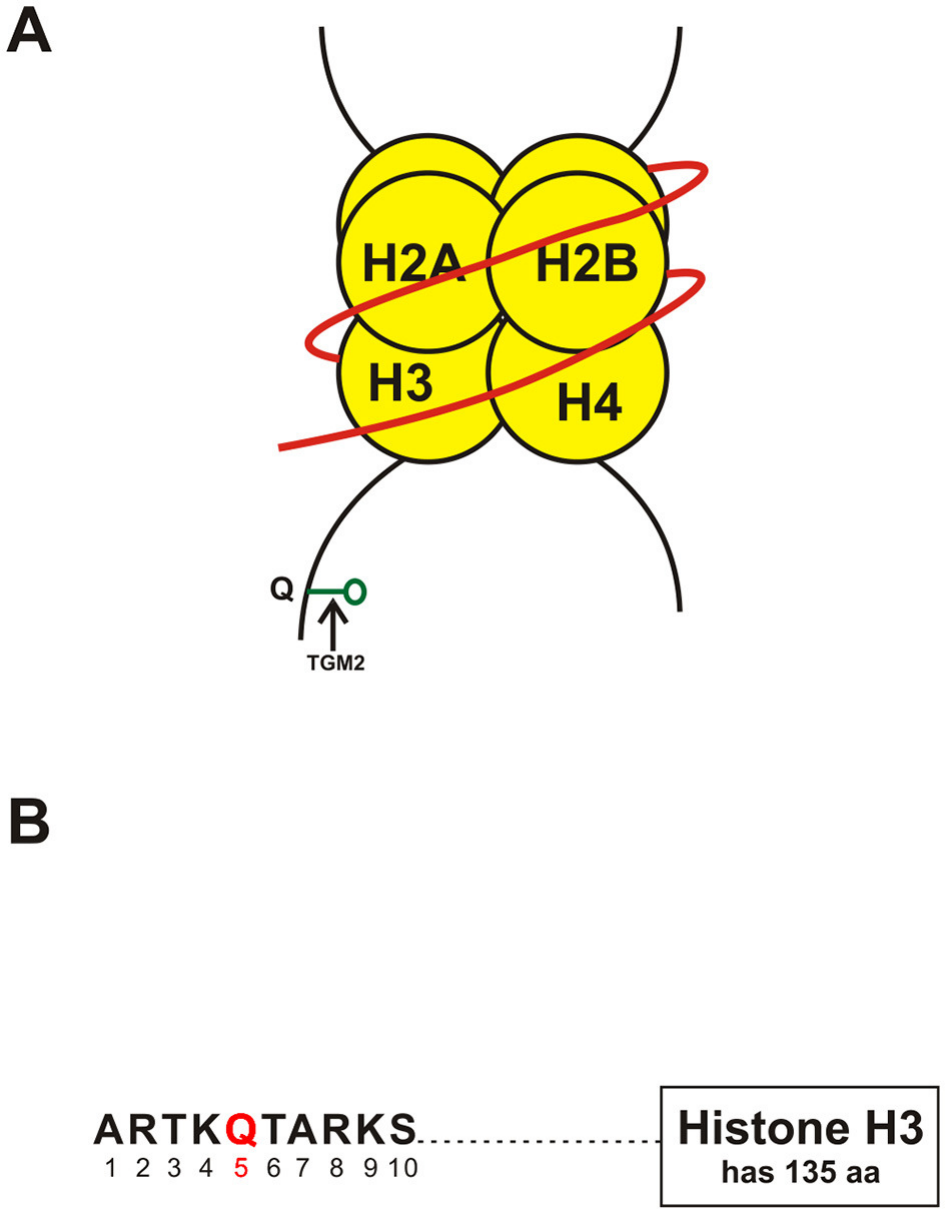
**(A)** Schematic showing monoaminylation at the glutamine 5 position on the tail of histone H3 (H3Q5) a possible new epigenetic mechanism in psychiatric disorders. The parts of the figure shown in yellow are the histones around which the DNA strand shown in red is coiled. TGM2, Transglutamine 2; Q, Glutamine (at position 5 of the tail of histone H3). **(B)** Schematic of a portion of the tail of histone H3 showing the presence of glutamine **(Q)** at position 5 (shown in red) of the histone tail. Abbreviations: aa, amino acids. A, alanine; R, arginine; S, serine; T, threonine; K, lysine; Q, glutamine.

A relatively new type of histone modification is monoaminylation (Figure 1). This was first shown in histones from chicken erythrocytes by Ballestar et al. (1996). In 2019, Ian Maze's group using sophisticated techniques and methodologies such as induced pluripotent stem cells, and preparation of truncated and recombinant histones, showed that serotonylation of glutamine happens at position 5 (Q5ser) on histone H3 in organisms which make serotonin (Farrelly et al., 2019). These workers showed that tissue TGM2 can catalyze the serotonylation of nuclesosomes having histone H3 trimethylated lysine 4 (H3K4me3) leading to the formation of H3K4me3Q5ser. The latter molecule shows a ubiquitous pattern of expression in mammalian tissues, especially in the brain and gastrointestinal tract, both of which are known to have large amounts of serotonin. Studies across the genome of human serotonergic neurons, murine developing brain, and cultured serotonergic cells suggest that H3K4me3Q5ser nucleosomes are increased in euchromatin, are responsive to differentiation of cells, and associated with increased gene expression (Farrelly et al., 2019). The Maze group also found that histone serotonylation increases general transcription factor IID (TFIID) binding to H3K4me3 and augments transcription (Girault, 2020). It was also found that cells which express ectopically a mutant of H3 which cannot be serotonylated show markedly changed expression of H3K3me3Q5ser-target loci, leading to differentiation deficits (Farrelly et al., 2019). Hence, Maze and colleagues inferred that there is a direct role for serotonin in permissive gene expression that is different from its role in neurotransmission and cell signaling.

The same group also showed that H3 serotonylation undergoes dynamic control during placental development, which corresponds to changes in gene expression that are known to affect important metabolic processes (Chan et al., 2024). The workers used transgenic mice to show that placental H3 serotonylation depends on serotonin uptake by the serotonin transporter (SERT). SERT deletion markedly decreases enrichment of H3 serotonylation across the placental genome, and affects neurodevelopmental networks of genes in early embryonic tissues of the brain. The authors inferred that these data suggest a new role for H3 serotonylation in gene transcription control in the placenta at the interface of maternal physiology and development of the fetal brain (Chan et al., 2024). However, more research is necessary to completely determine how the involvement of H3 serotonylation modulates tissue-specific functions. Moreover, a full catalog of monoaminylated proteins and their downstream effects on fetal neurodevelopment could explain how new and unusual monoamine mechanisms affect risk for neurodevelopmental disorders (Chan et al., 2024).

Histamine, like serotonin and dopamine, is involved in histone monoaminylation. The Maze group recently described H3 histaminylation which is also catalyzed by TGM2 and occurs at the same position on H3 (Q5). The authors used a H3Q5his antibody that they produced and validated in cells and brain tissues and showed that H3Q5his concentrations are increased at the sites of synthesis of histamine in the brain, namely, tuberomammillary nucleus (TMN), and which changes according to sleep-wake patterns. These changes were found to be affected by drugs that influence sleep patterns. The authors also found that TGM2 functions as a “writer” an “eraser” and a “rewriter” of monoaminylation of H3. The authors also found, based on several biochemical and structural studies, that H3Q5his reduces H3K4 methylation induced by the mixed lineage leukemia (MLL) complex (a family of methyltransferases) (Zheng et al., 2025).

## Monoaminylation of histones in psychiatric disorders

Monoaminylation of histones has also been investigated in psychiatric disorders. Maze's group has investigated histone serotonylation after stress and/or antidepressant exposures. The workers used a combination of genome-wide and biochemical studies in the dorsal raphe nucleus (DRN) of male and female mice subjected to chronic social defeat stress, and also in the DRN of patients with major depressive disorder (MDD). The workers studied the effect of exposure to stress and MDD on H3K4me3Q5ser and the association between H3K4me3Q5Ser and expression of genes related to MDD. Moreover, the authors investigated the stress-induced and MDD-linked control of H3K4me3Q5ser after antidepressant (fluoxetine) exposures, and used viral-mediated gene therapy in mice to decrease H3K4me3Q5ser levels in the DRN and study the impact on gene expression and behavior related to stress. They found that H3K4me3Q5ser influences stress-induced transcriptional plasticity. Chronically- stressed mice showed abnormal H3K4me3Q5ser activity in the DRN, with antidepressant- and viral-mediated disruption of this activity proving to be adequate to reduce stress-induced expression of genes and behavior. Corresponding patterns of H3K4me3Q5ser control were seen in patients with MDD on- as compared to off- antidepressant therapy at the time of death. Hence, the authors inferred that serotonin has a role in stress and antidepressant- associated transcription and behavior separate from its role in neurotransmission, and important in clinical MDD and its drug therapy (Al-Kachak et al., 2024).

Maze's group also discovered in 2020 that dopamine forms covalent bonds with histone H3 at glutamine residue 5 to form H3Q5dop (Lepack et al., 2020). Dopamine markedly influences learning and memory, reward, substance abuse, and motor function (Chan and Maze, 2020). Lepack et al. (2020) used a combination of liquid chromatography-mass spectrometry (LC-MS/MS) and enzymatic assays to show that like histone serotonylation, histone dopaminylation is catalyzed by TGM2 at H3Q5, and occurs in both isolated form (H3Q5dop) and in conjunction with K4 trimethylation (H3K4me3Q5dop) and correlates with regulation of transcription in the brain. In the ventral tegmental area (VTA), a brain region where dopamine is synthesized and takes part in behavioral reward, H3Q5dop concentrations were decreased in the rat as well as in human post-mortem brain following continuous use of cocaine. The authors found in the rat VTA that dysregulation of H3Q5dop depends on the timing of cocaine intake and withdrawal.

To better learn the functional effect of raised H3Q5dop during abstinence, Lepack and colleagues delivered dominant viral vectors into the VTA of rats to decrease H3Q5dop concentrations after exposure to cocaine. RNA-sequencing on virally—affected tissues showed that reduced H3Q5dop concentrations reverse the pattern of transcription that is normally observed during cocaine withdrawal. In addition, decreasing H3Q5dop prevented cocaine-seeking behavior in rats with abnormal drug—induced dopamine release into the nucleus accumbens, an important brain reward region that receives a dopaminergic nerve supply from the VTA. These findings suggested that dopamine has roles in mediating abnormal gene expression that are independent of its role in neurotransmission and that such roles are associated with occurrence of vulnerability to substance abuse relapse.

In addition to psychiatric disorders, monoaminylation has been noted in other disorders such as cancer (Li et al., 2024). Currently the most commonly monoaminylated amino acid residue on histone tails is glutamine 5 on the tail of histone H3 (Li et al., 2024), although it is possible that other amino acid residues are also targeted.

## Potential implications of histone monoaminylation for treating psychiatric disorders

Modified histones are known to be involved in the pathogenesis of psychiatric disorders (Panariello et al., 2022). However, these modifications involve the more well-known histone modifications such as histone acetylation (Panariello et al., 2022). The work discussed in the preceding sections above is the first to review histone monoaminylation in the pathogenesis of psychiatric disorders. This work is still very much in its infancy. However, there is evidence suggesting that histone monoaminylation is involved in the pathogenesis of psychiatric disorders. As discussed in the preceding sections, dopaminylation of histone H3 in the VTA plays a role in cocaine seeking (Lepack et al., 2020), and histone serotonylation in the DRN contributes to stress-and antidepressant (fluoxetine)-mediated gene expression and behavior (Al-Kachak et al., 2024). It is possible that in addition to the more well established histone modifications, histone monoaminylation is also involved in the pathogenesis of these and other psychiatric disorders.

There are several difficulties associated with the study and interpretation of histone modifications in the brain. They include (Park et al., 2022; Paul and Potter, 2024): the extreme complexity of the brain; temporal variations in histone modifications; variations of histone modifications according to brain tissue and cell type; the complexity of laboratory techniques required; and difficulties in determining whether findings are the cause or the effect of the disease process.

As mentioned, histone monoaminylation is catalyzed by TGM2. Inhibitors targeting TGM2 have been developed, encompassing both reversible and irreversible types, and are undergoing drug trials for use in conditions such as celiac sprue, neurodegenerative disorders, and cancer (Song et al., 2017; Badarau et al., 2015; Keillor and Johnson, 2021). The overall efficacy of present psychotropic drugs are arguably no better than the very first ones introduced more than fifty years ago, especially at improving functional outcomes of patients (Paul and Potter, 2024). Hence, new and better such drugs are urgently needed (Paul and Potter, 2024). The developments discussed in the current article, that is, monoaminylation of histones catalyzed by TGM2, are a possible new field of neuroscience for the development of psychotropic drugs. However, at present this is somewhat speculative, and to date, even preclinical (phase 0) studies have not been conducted in this area. Moreover, epigenetic mechanisms of gene expression are interconnected and if abnormalities of histone monoaminylation are present in a particular psychiatric patient, there are likely to be other epigenetic abnormalities as well (Aljabali et al., 2024), compounding the difficulties in finding new and effective drugs acting epigenetically for treating psychiatric disorders.
